# Examining the Role of Familiarity in the Perception of Depth

**DOI:** 10.3390/vision4020021

**Published:** 2020-04-02

**Authors:** Elizaveta Mischenko, Ippei Negishi, Elena S. Gorbunova, Tadamasa Sawada

**Affiliations:** 1 School of Psychology, National Research University Higher School of Economics, Moscow 101000, Russia; evmischenko_1@edu.hse.ru (E.M.); esgorbunova@hse.ru (E.S.G.); 2 Department of Media Informatics, Kanazawa Institute of Technology, Ishikawa 924-0838, Japan; negishi@neptune.kanazawa-it.ac.jp

**Keywords:** depth perception, pictorial depth cue, familiar size, familiarity, meta-analysis

## Abstract

Bishop Berkeley suggested that the distance of an object can be estimated if the object’s size is familiar to the observer. It has been suggested that humans can perceive the distance of the object by using such “familiarity” information, but most or many of the prior experiments that found an effect of familiarity were not designed to minimize or eliminate potential influences of: higher cognitive factors on the observers’ responses, or the influences of low-level image features in the visual stimuli used. We looked for the familiarity effect in two experiments conducted both in Russia and Japan. The visual stimuli used were images of three coins used in Russia and Japan. The participants’ depth perception was measured with a multiple-choice task testing the perceived depth-order of the coins. Our expectation was that any effect of “familiarity” on depth perception would only be observed with the coins of the participant’s country. We expected a substantial familiarity effect based on our meta-analysis of the “familiarity” effects observed in prior experiments. But, our results in both experiments showed that the familiarity effect was virtually zero. These findings suggest that the importance of a familiarity effect in depth perception should be reconsidered.

## 1. Introduction

We perceive the shapes of objects and their positions within a scene veridically based on visual information, and we can interact with them effectively in our everyday life. But, how the visual system can recover the 3D information contained in the scene and in the objects is actually a difficult problem. Several different explanatory mechanisms have been proposed to explain the veridicality of 3D perception. A few of these mechanisms are based entirely on geometrical optics, such as, binocular disparity, but most of the mechanisms use additional information to recover the 3D information contained within a scene. Some of these mechanisms rely on motor information possessed by the human body, for example, vergence eye movements, accommodation of the crystalline lens, and head movement. Other mechanisms use a priori constraints [1,2], and still others are based on memories of individual objects that were established during past experiences.

The role of memory in visual 3D perception has been discussed for centuries. An early highlight in these discussions includes Berkeley’s [3] realization that recovery of 3D information contained in a scene from the 2D information in its retinal image was problematic. He went on to suggest that early experience with the tactile perception of an object could be important for establishing the shape and distance of the object based only on visual information. For Berkeley, the physical shape, distance, and size of the object are initially perceived tactually and their relationship is learned from the experience with it. He suggested that the distance of an object can be estimated from the size of its retinal image if the physical size of the object is memorized.

Now consider a familiar object whose physical size is known to an observer on the basis of his/her past experience. In this study, being familiar means that an object commonly appears in our everyday life (see Supplemental Material for a list of the familiar objects that were used in prior studies). The present study does not consider any effect of an emotional attachment on any object (see [4,5,6,7,8] for studies and discussions about emotional effects of objects on the perceived distance of the object). It is theoretically possible to estimate the distance of this familiar object from its single 2D retinal image by comparing the size of the image with the familiar size of the object. We assume, for simplicity, that the object is planar, circular, and frontoparallel. The relationship between the size of the retinal image and the familiar size of the object is:

(1) $$D_{F}\sim \frac{{ø}_{F}}{\tan\alpha_{V}}$$

where *D_F_* is the distance, ${ø}_{F}$ is the diameter, and *α_V_* is the visual angle of the object. The visual system can use this relationship as a cue for estimating the distance of the object. Then, the depth between two familiar objects can be also estimated as the difference in the estimated distance between the objects. This hypothetical cue for perceiving both the distance and depth is referred to as the *familiarity* depth cue.

This familiarity cue may be the most intensively studied phenomenon considered when one is interested in studying how a higher cognitive factor affects the perception (see [9] for an early review). There have been more than 30 studies in the last 70 years reporting that this familiarity cue affects the perception of the distance within a scene and the depth of objects within it. This has led to familiarity being included in lists of the cues for depth perception in many articles (e.g., [10,11,12,13]) as well as in undergraduate textbooks of “Sensation & Perception” (e.g., [14,15]).

There are number of studies that reported the effects of other higher cognitive factors on perception (see [16] for a review), but the validity of many of these studies has been questioned because of methodological issues [16,17,18,19,20]. For example, the procedures in some of these experiments did not distinguish cognitive effects on the observers’ perception from the cognitive effects on their responses. Others were not well controlled so the cognitive factors reported could be explained by the operation of low-level image features of the stimuli. These criticisms can also be applied to many prior studies of the familiarity cue.

Many studies of the familiarity cue were not designed to minimize, or to eliminate, the effects of higher cognitive factors on the responses obtained (see [21,22,23,24] for exceptions). In these experiments, the perception of the distance and the depth was directly measured by using an adjustment task and an image of a familiar object was presented for an unlimited viewing duration when the observer made his judgment. This method allows the observer to engage in elaborate thinking about what the distance and depth might be while the stimulus is actually present [25,26,27,28,29,30,31]. For example, the judgment may be based on solving Equation (1) consciously rather than on the distance and depth perceived. This potential artifact encouraged us to use brief presentations of the visual stimuli to minimize the effect that thinking could have on the observer’s responses (p. 18 in [16]).

Systematic control of visual stimuli is particularly difficult when one wants to study the familiarity cue because the stimuli are images of objects that are familiar in our everyday life. These images often have unavoidably different low-level image features, and these low-level image features themselves could affect the perception of distance and depth. These features include size [32,33,34], spatial-frequency [35,36,37], contrast [38,39,40], color [41,42,43], and configuration [44,45]. These effects of low-level image features can be confounded with familiarity in measurements of distance and depth. For example, consider images of the golf and tennis balls that were used as visual stimuli in [46]. A golf ball is physically smaller than a tennis ball, so it was expected that the golf ball would be perceived to be closer than the tennis ball based on the familiarity cue if they appear with the same retinal size. But, the contrast of the golf ball’s retinal images was higher than the tennis ball’s. This contrast difference could be a confounding factor because the contrast also suggested that the golf ball was closer than the tennis ball. 

The relative sizes of two retinal images of an object, itself, can serve as a cue for the perception of the depth between the objects. This could also be an artifact in some studies of the familiarity cue [47,48,49,50,51,52]. These studies of the familiarity cue used multiple images of a single familiar object as visual stimuli [51,52,53,54]. Here, the distance and depth of the familiarity cue was controlled by changing the size of the images because the physical size of the familiar object was kept constant (see Equation (1)). When this is done, the depth could also be judged on the basis of the relative size of the images, across trials or within a trial. The authors of these studies tried to address this potential relative size artifact by using images of an unfamiliar object that had the same physical size as the familiar object did [22,55,56,57,58,59]. The effect of a familiarity cue on perception could be tested by comparing the perception of familiar and unfamiliar objects, but note that this control does not take into account other potential low-level image features on the perception of distance and depth.

**Figure 1:**
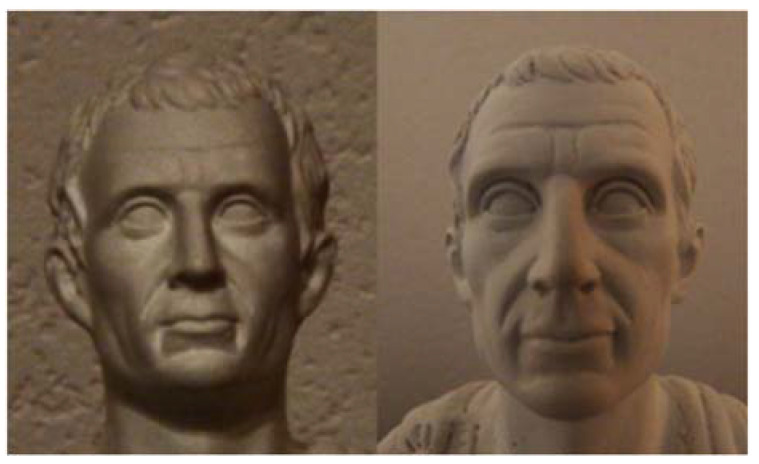
Two photographs of a single bust taken from two different distances. The head was about 5 cm vertically. The left photo was taken about 6 m from the bust, and the right photo was taken about 10 cm from the bust. The retinal projections of these two photos only represent the original bust when they are viewed from their correct centers of projection.

Another problem encountered in the visual stimuli used in studies of the familiarity cue is in the retinal projection of the image of a volumetric object. The relationship between retinal projections of an object at two different distances cannot be represented simply by scaling the image of the object unless the object is planar and frontoparallel (Figure 1). A number of studies tried to control the distance suggested by a familiarity cue by merely scaling a single image of the volumetric object (e.g., [47,60,61]). But, retinal projections of these scaled images cannot be a projection of the original object, and the perceived shapes of the object based on these retinal projections can be different from one another [1,62]. Furthermore, the perceived distance of the object can be affected by the difference between the two retinal projections and between the two shapes perceived.

In the present study, we tested the effect of a familiarity cue on the perception of depth. Potential artifacts in our visual stimuli were addressed by comparing two groups of participants who were familiar with different sets of objects. Participants were recruited in Russia and in Japan and the visual stimuli were images of both Russian and Japanese coins. Participants were familiar with the coins from their own country but not with the coins from the other country. The participants’ depth perception was measured with a multiple-choice task in which the participant’s ability to perceive the depth-order of the coins was assessed. Depth-order was indicated by a familiarity cue. If depth could be perceived on the basis of a familiarity cue, both groups of participants would be able to perform the tasks reliably only with the coins of their own country, but not with the coins from the other country. These arrangements made it possible for us to study familiarity without any artifacts inherent in the visual stimuli, themselves.

## 2. Meta-Analysis

We compared effects of familiarity on perceived distance and on perceived depth by conducting a meta-analysis of published studies concerned with familiarity effects. These effects were analyzed separately and the results of the two analyses were compared. We first collected 35 published empirical studies of the familiarity effects (see Supplemental Material). Samples for the two meta-analyses were taken from these studies based on criteria specified.

### 2.1. Distance from Familiarity

We started by evaluating the familiarity effect on distance perception. Samples for this analysis were collected from nine studies [21,26,46,63,64,65,66,67,68] (see also Supplemental Material) that were selected by using the following criteria: (a1) Multiple familiar objects with different physical size were shown across trials in each session; (a2) the image of a single object was presented monocularly at eye-height as the visual stimulus in each trial; (a3) the observer reported the perceived distance of the object by using a direct method; (a4) the distance response was represented metrically; and (a5) the physical size of the object was given or could be specified.

**Figure 2:**
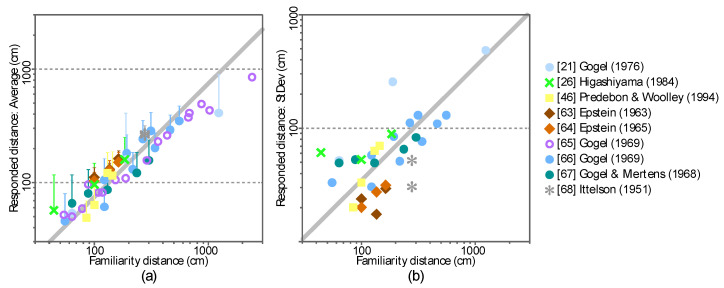
Average and standard deviation of the distance responses in the nine published studies. (**a**) The abscissa shows the familiarity distance and the ordinate shows the average of the distance responses averaged across participants in each study. The error bars show the standard deviations across the participants. (**b**) The standard deviation of the distance responses is plotted separately. The abscissa shows the familiarity distance and the ordinate shows the standard deviation of the distance responses. Note that only the average of responses was reported in [65]. Data from [65] is plotted only in (**a**) (open circles) but not in (**b**).

Figure 2a shows the averages of the distance responses across participants in the nine published studies. The ordinate represents the average response and the abscissa represents the familiarity distance. The familiarity distance was computed by using Equation (1) from the visual angles of the visual stimuli that were images of familiar objects and the physical size of the familiar objects. The error bars represent the standard deviations of the depth responses. The standard deviations were also plotted in Figure 2b. The ordinate represents the standard deviation and the abscissas represent the familiarity distance.

The average distance response (*R* = 0.96, *p* < 2.2 × 10^−16^) and the standard deviation (*R* = 0.85, *p* = 3.2 × 10^−10^) are positively correlated with the familiarity distance. These trends can be characterized as follows:

(2) $$\{\begin{array}{c} \mu_{d}=\alpha_{d}d\\ \sigma_{d}=\beta_{d}d \end{array}$$

where *α**_d_*** and *β**_d_*** are constants, d is the familiar distance, and *μ_d_* and *σ_d_* are the average and standard deviation of the distance responses. The constants *α_d_* and *β**_d_*** in this equation were determined as *α**_d_*** = 0.7471 and *β**_d_*** = 0.3499 by using the least squares method.

### 2.2. Depth from Familiarity

We next evaluated the familiarity effect on the perception of the depth between two objects. Samples for this analysis were taken from two studies [33,67] (see also Supplemental Material) that were selected by using the following criteria: (b1) They had multiple familiar objects with different physical sizes that were shown across the trials in each session; (b2) the images of two objects were presented together monocularly at eye-height served as the visual stimulus in each trial; (b3) the observer used a direct method to report the perceived depth between the objects; (b4) the depth response was represented metrically; (b5) the physical size of the object was given or could be specified; and (b6) the familiarity depth (the difference between the familiarity distance of the two objects) was not zero.

**Figure 3:**
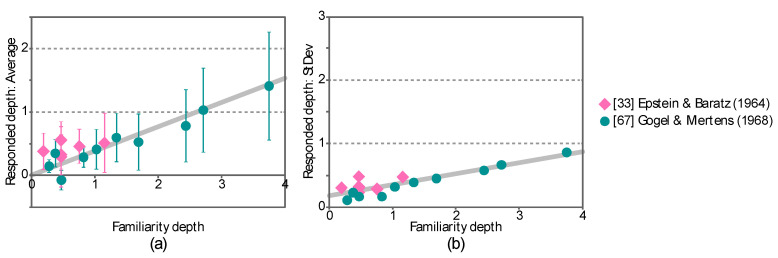
Standardized average and standardized standard deviation of the depth responses in the two published studies. (**a**) The abscissa shows the standardized familiarity depth and the ordinate shows the standardized average of the depth responses averaged across participants in each study. The error bars show the standardized standard deviations across the participants. (**b**) The standard deviation of the distance responses is plotted separately. The abscissa shows the standardized familiarity distance and the ordinate shows the standardized standard deviation of the depth responses.

Figure 3a shows the averages of the depth responses across the participants in the two published studies. Note that the averages and standard deviation of the response and familiarity depth were standardized by dividing them by the familiarity distance of the closer object. The ordinate represents the standardized average response and the abscissa represents the standardized familiarity depth. The error bars represent the standardized standard deviations of the depth responses. The standardized standard deviations were also plotted in Figure 3b. The ordinate represents the standardized standard deviation and the abscissas represent the standardized familiarity depth.

The standardized average response (*R* = 0.91, *p* = 1.5 × 10^−9^) and the standardized standard deviation (*R* = 0.87, *p* = 7.5 × 10^−8^) are positively correlated with the familiarity distance. These trends can be characterized as follows:

(3) $$\{\begin{array}{c} \mu_{AB}=\alpha_{AB}\left(d_{B}-d_{A}\right)\\ \sigma_{AB}=\beta_{AB}\left(d_{B}-d_{A}\right)+\kappa_{AB}d_{A} \end{array}$$

where *α_AB_*, *β_AB_*, and *κ_AB_* are constants, *d*_A_ and *d*_B_ are the familiarity distance of the two objects (*d*_A_ ≤ *d*_B_), and *μ_AB_* and *σ_AB_* are the average and standard deviation of the depth responses. The constants *α_AB_*, *β_AB_*, and *κ_AB_* in this equation were determined as *α_AB_* = 0.3840, *β**_AB_*** = 0.1762, and *κ**_AB_*** = 0.1719 by using the least squares method.

### 2.3. Comparing Distance and Depth from Familiarity

The familiarity effects on the perception of distance and depth were compared on the basis of the results of the two meta-analyses.

Consider the depth response. The relationship between the depth response and the familiarity depth can be characterized by Equation (3). Note that depth is the difference in the distance of the two objects. The difference between the distance responses of the individual objects can be computed from Equation (2):

(4) $$\{\begin{array}{c} {\dot{\mu }}_{AB}=\alpha_{d}\left(d_{B}-d_{A}\right)\\ {\dot{\sigma }}_{AB}=d_{A}\beta_{d}\sqrt{1+{\left(1+\left(d_{B}-d_{A}\right)\right)}^{2}} \end{array}$$

where ${\dot{\mu }}_{AB}$ and ${\dot{\sigma }}_{AB}$ are the average and standard deviation of the difference of the distance responses and *d*_A_ ≤ *d*_B_. This equation represents hypothetical responses describing the depth when the depth responses are based on the distance perception of individual objects.

**Figure 4:**
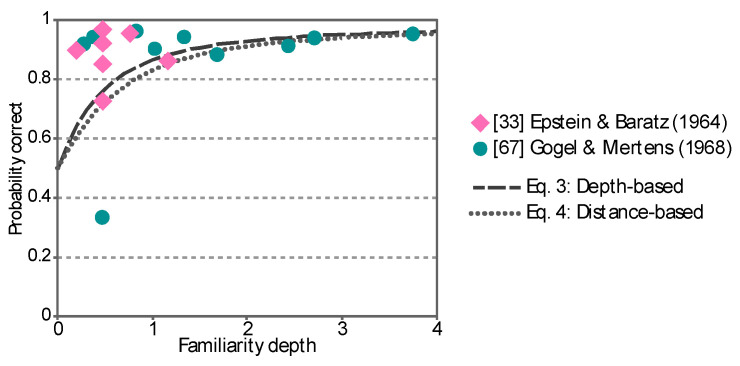
Computed probability for a correct depth order response based on: (i) Depth responses (Equation (3)), (ii) the difference of the distance responses (Equation (4)), and (iii) individual samples used in the meta-analysis about the published studies of the familiarity effects on the depth perception [33,67] (Figure 3). The ordinate represents the computed probability correct and the abscissa represents the standardized familiarity depth. The symbols represent the bases used for the computation of the probability correct.

The probability that an observer’s responses are correct (*p_correct_*) and incorrect (*p_error_*) depth orders between the objects can be computed as follows:

(5) $$\{\begin{array}{c} p_{correct}=\int_{0}^{\infty }\frac{1}{\sigma \sqrt{2}}e^{\frac{-{\left(x-\mu \right)}^{2}}{2\sigma^{2}}}dx\\ p_{error}=\int_{-\infty }^{0}\frac{1}{\sigma \sqrt{2}}e^{\frac{-{\left(x-\mu \right)}^{2}}{2\sigma^{2}}}dx \end{array}$$

where *μ* and *σ* are average and standard deviation of depth responses. Figure 4 shows the probability of the correct depth order when: (i) It is computed on the basis of the depth responses (Equation (3)), (ii) on the difference of the distance responses (Equation (4)), and (iii) on individual samples used in the meta-analysis of the studies about the familiarity effects on the depth perception [33,67] (Figure 3). The ordinate represents the computed probability correct and the abscissa represents the standardized familiarity depth. The symbols represent the bases used for the computation of the probability correct. The probability correct was higher (i) when it was based on the depth responses (Equation (3)) than (ii) when it was based on the difference between the distance responses (Equation (4)). This suggests that the observer can judge the depth between the objects more reliably than he/she can judge the distance of the individual objects. Note that the psychophysical experiments in this study were designed to test the perception of depth. Equation (3) was used for the power analysis of the experiments.

## 3. Psychophysical Experiments

### 3.1. Methods

#### 3.1.1. Participants

Forty Russian students and forty Japanese students participated in two experiments named 1 and 2 based on the order in which they were performed. The Russian participants were undergraduate students in the Department of Psychology at the National Research University Higher School of Economics. They had never visited Japan. The Japanese participants were undergraduate students in the Department of Media Informatics at the Kanazawa Institute of Technology. They had never visited Russia. Both groups participated voluntarily and all participants were naive with respect to the purpose of these experiments. Both groups were tested individually in Russia and in Japan. Participants were at least 18 years old and had normal, or corrected-to-normal, vision.

The experiments reported in this study were conducted in accordance with the Code of Ethics of the World Medical Association (Declaration of Helsinki) and approved by the institutional review board (IRB: “Stereo 3D perception” at the National Research University Higher School of Economics; No. 18278 at the Kanazawa Institute of Technology). We obtained written consent from all participants prior to the experiments.

#### 3.1.2. Apparatus

Two very similar apparatuses were set up in Russia and in Japan. Visual stimuli were displayed on a computer-controlled display. The resolution of the Russian display was 1920 × 1200 pixels, and its size was 33.2 cm × 20.7 cm with a 60 Hz refresh rate. The resolution of the Japanese display was 2560 × 1440 pixels, and its size was 59.7 cm × 33.5 cm with a 60 Hz refresh rate. Each apparatus had two circular apertures. The display was viewed through a 2-cm diameter aperture that was 66 cm from the display. The other aperture, which had a 10-cm diameter, was located 30 cm from the display. It prevented the participants from seeing the rectangular boundary of the display. The visual angle of the visible region of both displays was 15.8 degrees. The centers of the apertures were on a line that was normal to the display. The experiments were conducted in completely dark rooms.

#### 3.1.3. Stimuli

**Figure 5:**
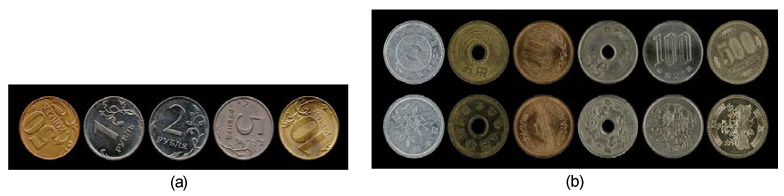
Images of the (**a**) Russian coins and (**b**) Japanese coins.

Images of eleven types of the coins were used to compose the test visual stimuli. Five types of Russian coins (50 kopeks and 1, 2, 5, and 10 Rubles) and six types of Japanese coins (1, 5, 10, 50, 100, 500 Yen) were used (Figure 5, Table 1). Twenty examples of each type of Russian coin were sampled and the sides that had numbers were scanned with the coins in random orientations (Russian Ruble coins have the same designs on both sides). This generated a total of 100 images. Ten examples of each type of Japanese coin were sampled and both sides were scanned with the coins in random orientations. This generated a total of 120 images. These images, selected randomly, make up the visual stimuli that will be used across the trials and across the participants in the proposed experiments. The images of the coins were scaled to make them 1.95 degrees of visual angle (a 2.25 cm diameter in the display). The size of the images was 130 × 130 pixels for the Russian display and 97 × 97 pixels for the Japanese display.

The visual stimulus used in Experiment 1 was static. It contained images of three coins. These images were aligned horizontally. The centers of the images were positioned: (i) At the center of the display and (ii) 2.25 degrees (2.59 cm) to the left and right of the center. These images are referred to as Coins-A, -B, and -C. Each test stimulus was presented for 300 ms. The visual stimulus used in Experiment 2 was dynamic. It was a temporal sequence of the images of three coins presented at the center of the screen. These images are referred to as Coins-A, -B, and -C in the temporal pattern. Each image was shown for 200 ms.

The coins were expected to be sufficiently familiar in each of their countries because they were commonly used by students both in Moscow, Russia and in Kanazawa, Japan when the experiments were conducted (2018). Images of these coins had also been used in several prior studies for testing depth perception based on familiarity [33,63,64,67]. Note that an effect of familiarity on the depth perception had been reported when objects less common in our everyday life were used (e.g., playing cards [21,25,51,52,53,54,55,57,65,69,70,71] and sports balls [23,24,46,60,61,72]; see Supplemental Material for a list of familiar objects used in the past studies).

#### 3.1.4. Procedure

**Figure 6:**
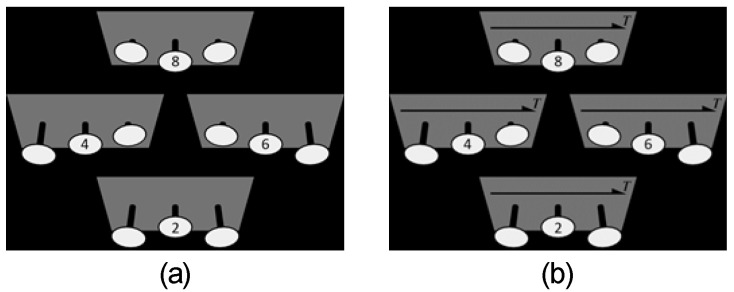
Images used to respond in Experiments 1 (**a**) and 2 (**b**). The four panels in each image show the four alternative choices available in the task. Ellipses in each panel represent Coins-A, -B, and -C from left to right. Numbers in the ellipses of Coin-B show the keys for the response.

Each trial began with fixation of a small cross at the center of the display for 500 ms. It was followed by presentation of a test visual stimulus. A noise pattern was then shown for 500 ms to mask the persistence of the test stimulus. An image the participant could use for responding was shown (Figure 6) until the participant responded by reporting the perceived depth-order of the coins in the 4-alternative forced choice task (4AFC). This response was made by pressing a key: Coin-B was the farthest (key-2), Coin-B was farther than Coin-A and closer than Coin-C (key-4), Coin-B was farther than Coin-C and closer than Coin-A (key-6), and Coin-B was the closest (key-8). The participant was clearly instructed to respond based on their perception of depth but not on any elaborate thinking [25,26,27,28,29,30,31].

All participants were informed before the experiments that the images of the Russian and Japanese coins would be shown in the display.

Each experiment had eight trials with both the Russian and Japanese coins. The order of these 16 trials was randomized within each session of the experiment. The sessions were controlled by using *PsychoPy2* [73]. Note that each visual test stimulus had images of three coins. The types of coins used as stimuli were specified for all 16 trials (Table 2). Coin types A and C were swapped randomly. Such compositions were used in both Experiments 1 and 2 across all participants. The images of the coins composing the stimuli were chosen randomly from scanned images of each type of coin. This randomization was done by using GNU Octave (https://www.gnu.org/software/octave/). This was done for all of the participants before the experiments and saved as text files.

Both Experiments 1 and 2 were pre-registered in Open-Science-Framework (https://osf.io/n5czq).

#### 3.1.5. Power-Analysis

The participant responded by making a 4AFC about the depth-order of the coins in the visual stimulus. The participant’s performance is expected to be at chance (25%) if s/he is not familiar with the coins. If the participant *is* familiar with the coins, the expected performance can be computed as follows.

The specific task in a given trial can be decomposed into two independent decisions about the depth-orders between Coins-A and -B and between Coins-B and -C. The expected perception between Coins-A and -B is represented as a Gaussian normal distribution whose mean *μ*_AB_ and standard deviation *σ*_AB_ can be computed from their familiarity distance *d*_A_ and *d*_B_ (Table 1) and Equation (3). From *μ*_AB_ and *σ*_AB_, the expected probability *p*_AB_ of the correct decision being made about the binary decision concerning the depth-order of Coins-A and -B can be computed with Equation (5). The expected probability *p*_BC_ of the correct depth-order of Coins-B and -C can be computed in the same way. Once this is done, the expected probability *p*_ABC_ of obtaining the correct 4AFC response can be computed as *p*_ABC_ = *p*_AB_*p*_BC_. This expected probability was computed for all compositions of the coins used in the experiments (Table 2).

The power of Experiments 1 and 2 were estimated in a simulation experiment by running a Monte Carlo simulation. Two types of model observers were defined, one to emulate the Russian and one to emulate the Japanese participants. In the 4AFC task, the Russian model observer performed at chance (*p*_ABC_ = 0.25) with the Japanese coins and performed according to the expected probability (Table 2) with the Russian coins. In the 4AFC task, the Japanese model observer performed at chance with the Russian coins and performed according to the expected probability with the Japanese coins. Each response of the model was determined as follows:

(6) $$\mathrm{response}=\{\begin{array}{c} \mathrm{correct}\;\mathrm{if}\;p_{ABC}>rand\\ \mathrm{wrong}\;\mathrm{otherwise} \end{array}$$

where *rand* is a random number generator using white noise between 0 and 1 (0 < *rand* < 1).

Let *N*_1/2_ be number of Russian and Japanese models. In the simulation experiment, the responses of *N*_1/2_ Russian models and of *N*_1/2_ Japanese models were collected for all compositions of the coins. The results of the simulation experiment were analyzed in the same way as the behavioral results.

The first step in the analysis was a 2-factor mixed-design ANOVA with repeated-measures on only one factor [74], specifically, two types of model observers (a between-subject factor) and two countries of coins (a within-subject factor). If the interaction between the model types and the countries of coins was significant (*p* < 0.01), a post-hoc test was conducted to examine whether the interaction was consistent with the expected effect of familiarity serving as a depth cue. In the post-hoc test, the difference in the performance between the two countries of coins was computed for each model observer and the computed difference was then compared between the two model types by using a two-sample one-tailed t-test with equal variance. A one-tailed test was used because the expected effect of familiarity is directional. If the post-hoc test was significant (*p* < 0.01), it was considered to support a familiarity effect.

The simulation experiment was repeated 1000 times for each given value of *N*_1/2_ to compute the probability that a familiarity effect had been observed. This process was repeated ten times and the average probability was computed. When *N*_1/2_ was set to 40, the average probability that a familiarity effect had been observed was about 90% (Table 3) (Note that the number of Russian and Japanese participants was registered to be *N*_1/2_ = 40 (https://osf.io/n5czq), but the pre-registration also reported that the estimated probability for observing a familiarity effect with *N*_1/2_ = 50. This inconsistency of *N*_1/2_ is our mistake.)

### 3.2. Results

**Figure 7:**
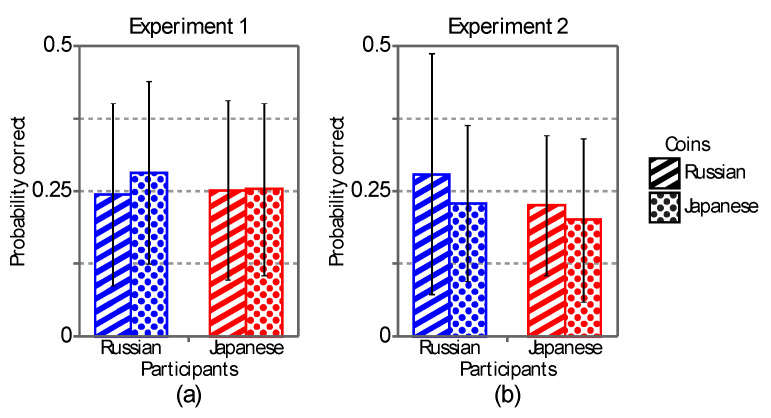
The results obtained in Experiments 1 (**a**) and 2 (**b**). The ordinate shows the probability of the correct 4AFC response, and the symbols show the four conditions (2 × 2) of the experiments: the two groups of participants and the two countries of the coins. Error bars show the standard deviations calculated from 40 participants.

Figure 7 shows results of the two psychophysical experiments averaged across the participants. The ordinate shows the probability of the correct 4AFC response. The symbols show the four conditions (2 × 2) in the experiments: the two groups of participants from Russia and from Japan (a between-subject factor) and the two countries of the coins (a within-subject factor). The results of the experiments were analyzed individually by using a 2-factor mixed-design ANOVA with repeated-measures on only one factor. The results of the ANOVA did not show any significant main effect in Experiments 1 and 2 (Table 4).

**Figure 8:**
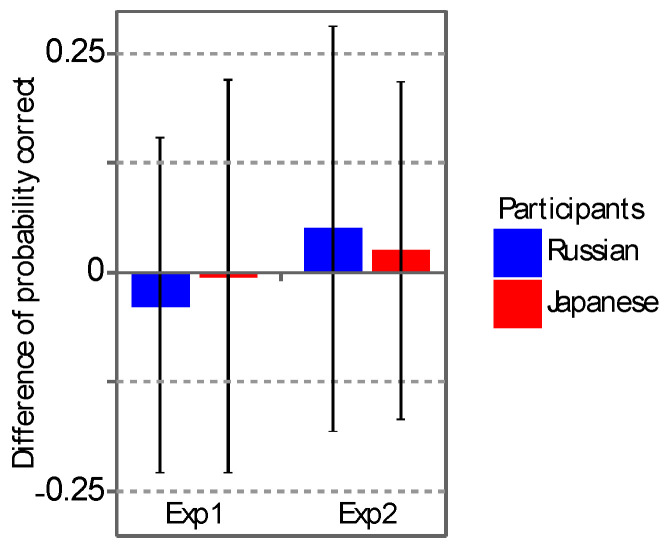
The difference in the performance between the two countries of coins for each group of participants in Experiments 1 and 2. The ordinate shows the difference, and the symbols show the groups of participants. Error bars show the standard deviations calculated from 40 participants.

Note that we expected a familiarity effect on depth perception to be observed as an interaction between the groups of participants and the countries of coins in Experiments 1 and 2. But, there was none (Table 4). The failure to find a familiarity effect was examined in detail. Namely, the difference in the performance between the two countries of coins was computed for each participant (Figure 8). This difference was then compared between the groups of participants by using a two-sample one-tailed t-test with equal variance (*t*_78_ = −0.7333, *p* = 0.2328 in Experiment 1; *t*_78_ = −0.5232, *p* = 0.3012 in Experiment 2). Scaled JZS Bayes factors for the null hypothesis [75] were also computed from the results of the t-test (*B*_01_ = 3.406 in Experiment 1 and *B*_01_ = 3.820 in Experiment 2). The computed Bayes factors (“substantial”, see [76]) show that any effect of familiarity on depth perception was either zero or, at most, very small (see also [77] for a discussion of supporting a null hypothesis based on Bayes factors).

## 4. Discussion

We tested the effect of a familiarity cue on the perception of depth in two psychophysical experiments. The effect size of the familiarity cue was estimated on the basis of a meta-analysis of past studies. Our experiments were designed to make their power high enough to be observed with the estimated effect obtained from our meta-analysis. However, the results of our experiments did not show any effect of a familiarity cue, not even a predicted trend. Our experiments clearly show that familiarity, studied, for the first time, without any obvious confounds, does not serve as a cue for depth.

Recall that our experiments were explicitly designed to minimize, or eliminate, confounding factors that were present in all prior experiments known to the authors. Our observers only saw images of familiar objects for short durations to prevent them from contemplating alternative choices before responding [16]. The durations were expected to be long enough for the familiar objects to be recognized on the basis of past studies (e.g., [78]). Furthermore, the images of Russian and Japanese coins we used as visual stimuli, as well as the participants were recruited in Russia and in Japan. This cross-cultural design allowed us to eliminate any effect of low-level image features (see the Introduction and Methods for details). On the other hand, the short viewing duration also limits generalization of our results. The human cognitive system may require a longer viewing duration to retrieve memorized size of the familiar objects. It is also possible that the effect of the familiarity cue on the perception of depth may require a longer viewing duration. We would need a very different experimental design to address the viewing duration issue.

Studying any effect of familiarity on visual perception is challenging because the systematic control of familiarity is difficult. Also, the failure to observe any familiarity effect can always be explained away simply by claiming that the observers did not have enough familiarity with the objects used as the stimuli. It can be also argued that the observers were familiar with the objects but not with their specific size because the observers were exposed to images and videos of the objects that had a wide variety of sizes. This kind of often-used explanation is likely to have introduced a publication bias [6,79,80,81,82] into studies on the effect of familiarity on the perception of depth and distance, as well as into studies on the effect of familiarity on the perception of other modalities (e.g., size, color). The conclusion we draw from our study is that all prior studies purporting to support a familiarity effect should be reviewed carefully and then reconsidered [16,83].

## Figures and Tables

**Table 1:** Types of coins used in this study and their physical diameters. The right most column shows their distance based on familiarity as a depth cue. The familiarity distance was computed from the physical diameters of the coins and the visual angle of their images (1.95 degrees) by using Equation (1).

Coin	Diameter (cm)	Familiarity Distance (cm)
50 kopeks	1.95	57.2
1 Ruble	2.05	60.1
2 Rubles	2.30	67.5
5 Rubles	2.50	73.3
10 Rubles	2.20	64.5
1 Yen	2.00	58.7
5 Yen	2.20	64.5
10 Yen	2.35	68.9
50 Yen	2.10	61.6
100 Yen	2.26	66.3
500 Yen	2.65	77.7

**Table 2:** Compositions of the visual test stimuli used in Experiments 1 and 2. The three columns on the left show the types of coins whose images were used as stimuli. The three columns on the right show the expected probability that a participant could correctly judge the depth-order between Coins-A and -B, the depth-order between Coins-B and -C, and both of these depth-orders. The expected probability was computed from the familiarity distances of the coins (Table 1) by using Equations (3) and (5) (see power-analysis).

Coin-A	Coin-B	Coin-C	Expected Probability Correct		
			A vs. B (pAB)	B vs. C (pBC)	Both (pABC)
2 Rubles	1 Ruble	10 Rubles	0.632873	0.560416	0.354672
2 Rubles	1 Ruble	5 Rubles	0.595654	0.655508	0.390456
10 Rubles	2 Rubles	50 kopeks	0.538637	0.632543	0.340711
50 kopeks	10 Rubles	1 Ruble	0.5999	0.560416	0.336193
5 Rubles	2 Rubles	50 kopeks	0.570765	0.632543	0.361033
5 Rubles	2 Rubles	1 Ruble	0.570765	0.595654	0.339978
5 Rubles	10 Rubles	50 kopeks	0.60535	0.5999	0.363149
5 Rubles	10 Rubles	1 Ruble	0.60535	0.560416	0.339247
100 Yen	50 Yen	500 Yen	0.56271	0.677663	0.381328
100 Yen	50 Yen	10 Yen	0.56271	0.593662	0.334059
1 Yen	10 Yen	5 Yen	0.629835	0.556592	0.350561
50 Yen	10 Yen	5 Yen	0.593662	0.556592	0.330428
10 Yen	5 Yen	1 Yen	0.556592	0.58027	0.322974
500 Yen	50 Yen	5 Yen	0.677663	0.540386	0.3662
500 Yen	10 Yen	50 Yen	0.599532	0.593662	0.355919
500 Yen	100 Yen	50 Yen	0.628356	0.56271	0.353582

**Table 3:** The results of the power analysis. The average and standard deviation of the probability that a familiarity effect had been observed were computed from the ten sessions of the 1000 simulation experiments. Two different levels of significance were used (1% and 5%). The expected effect on the perception was computed based on Equations (3) and (4).

	Equation (3): Depth-Based	Equation (4): Distance-Based
*p* < 5%	0.9743 ± 0.0048	0.8189 ± 0.0106
*p* < 1%	0.9072 ± 0.0076	0.6031 ± 0.0146

**Table 4:** Results of the statistical analysis in Experiments 1 and 2. Their psychophysical results were analyzed by using a 2-factor mixed-design ANOVA with repeated-measures on only one factor.

Experiment 1		
The groups of participants	*F*_1,78_ = 0.1856	*p* = 0.6678
The countries of coins	*F*_1,78_ = 0.7511	*p* = 0.3888
Interaction	*F*_1,78_ = 0.5378	*p* = 0.4656
Experiment 2		
The groups of participants	*F*_1,78_ = 2.6417	*p* = 0.1081
The countries of coins	*F*_1,78_ = 2.4632	*p* = 0.1206
Interaction	*F*_1,78_ = 0.2737	*p* = 0.6024

## Supplementary Materials

The following is available online at https://www.mdpi.com/2411-5150/4/2/21/s1, Data S1: Meta-Analysis.

## References

[1] Pizlo Z. (2008). 3D Shape: Its Unique Place in Visual Perception.

[2] Pizlo Z., Li Y., Sawada T., Steinman R.M. (2014). Making a Machine That Sees Like US.

[3] Berkeley G. (1709). An Essay Towards a New Theory of Vision.

[4] Balcetis E., Dunning D. (2010). Wishful seeing: More desired objects are seen as closer. Psychol. Sci..

[5] Dion K.L., Dion K.K. (1976). The honi phenomenon revisited: Factors underlying the resistance fo perceptual distortion of one’s partner. J. Personal. Soc. Psychol..

[6] Francis G. (2012). The same old new look: Publication bias in a study of wishful seeing. i-Perception.

[7] Ong J., Luck W.J., Olson H.A. (1980). Reliability, sex difference, and honi phenomenon in a distorted room. Percept. Mot. Ski..

[8] Wittreich W.J. (1952). The Honi phenomenon: A case of selective perceptual distortion. J. Abnorm. Psychol. Soc. Psychol..

[9] Gogel W.C. (1964). Size cue to visually perceived distance. Psychol. Bull..

[10] Chandler D., Munday R., Chandler D., Munday R. (2011). Pictorial Depth Cues. A Dictionary of Media and Communication.

[11] Howard I.P. (2012). Perceiving in Depth. Other mechanisms of Depth Perception.

[12] Sedgwick H.A., Boff K.R., Kaufman L.I., Thomas J.P. (1986). Space perception. Handbook of Perception and Human Performance, Volume 1: Sensory Processes and Perception.

[13] Depth Perception [Wiki on the Internet]. https://en.wikipedia.org/wiki/Depth_perception.

[14] Goldstein E.B., Brockmole J. (2016). Sensation & Perception.

[15] Schwartz B.L., Krantz J.H. (2017). Sensation & Perception.

[16] Firestone C., Scholl B.J. (2016). Cognition does not affect perception: Evaluating the evidence for top-down effects. Behav. Brain Sci..

[17] Bruner J.S., Bruner J.S. (1973). The functions of perceiving: New look retrospect. Beyond the Information Given: Studies in the Psychology of Knowing.

[18] Durgin F.H., Li Z., Shapiro A.G., Todorović D. (2017). Why do hills look so steep?. Oxford Compendium of Visual Illusions.

[19] Merriman W.E., Moore Z., Granrud C.E. (2010). Children’s strategic compensation for size underconstancy: Dependence on distance and relation to reasoning ability. Vis. Cogn..

[20] Granrud C.E., Hatfield G., Allred S. (2012). Judging the size of a distant object: Strategy use by children and adults. Visual Experience: Sensation, Cognition, and Constancy.

[21] Gogel W.C. (1976). An indirect method of measuring perceived distance from familiar size. Percept. Psychophys..

[22] Hands P., Khushu A., Read J.C.A. (2014). Interaction between size and disparity cues in distance judgements. Proceedings of the International Conference on 3D Imaging (IC3D).

[23] Martín A., Chambeaud J.G., Barraza J.F. (2015). The effect of object familiarity on the perception of motion. J. Exp. Psychol. Hum. Percept. Perform..

[24] Martín A., Décima A.P., Barraza J.F. (2017). Perception of speed, distance, and TTC of familiar objects. Psychol. Neurosci..

[25] Predebon J. (1992). The role of instructions and familiar size in absolute judgments of size and distance. Percept. Psychophys..

[26] Higashiyama A. (1984). The effects of familiar size on judgments of size and distance: An interaction of viewing attitude with spatial cues. Percept. Psychophys..

[27] Baird J.C. (1963). Retinal and assumed size cues as determinants of size and distance perception. J. Exp. Psychol..

[28] Durgin F.H., Baird J.A., Greenburg M., Russell R., Shaughnessy K., Waymouth S. (2009). Who is being deceived? The experimental demands of wearing a backpack. Psychon. Bull. Rev..

[29] Durgin F.H., Klein B., Spiegel A., Strawser C.J., Williams M. (2012). The social psychology of perception experiments: Hills, backpacks, glucose and the problem of generalizability. J. Exp. Psychol. Hum. Percept. Perform..

[30] Predebon G.M., Wenderoth P.M., Curthoys I.A. (1974). The effects of instructions and distance on judgments of off-size familiar objects under natural viewing conditions. Am. J. Psychol..

[31] Woods A.J., Philbeck J.W., Danoff J.V. (2009). The various perceptions of distance: An alternative view of how effort affects distance judgments. J. Exp. Psychol. Hum. Percept. Perform..

[32] Burnham D.K. (1983). Apparent relative size in the judgement of apparent distance. Perception.

[33] Epstein W., Baratz S.S. (1964). Relative size in isolation as a stimulus for relative perceived distance. J. Exp. Psychol..

[34] Epstein W., Franklin S. (1965). Some conditions of the effect of relative size on perceived relative distance. Am. J. Psychol..

[35] Brown J.M., Weisstein N. (1988). A spatial frequency effect on perceived depth. Percept. Psychophys..

[36] Marshall J.A., Ariely D., Rolland J.P. (1996). Occlusion edge blur: A cue to relative visual depth. J. Opt. Soc. Am. A.

[37] Mather G. (1997). The use of image blur as a depth cue. Perception.

[38] O’shea R.P., Blackburn S.G., Ono H. (1994). Contrast as a depth cue. Vis. Res..

[39] O’shea R.P., Sekuler R. (1997). Blur and contrast as pictorial depth cues. Perception.

[40] Tai N., Inanici M. (2012). Luminance contrast as depth cue: Investigation and design applications. Comput. Des. Appl..

[41] Egusa H. (1983). Effects of brightness, hue, and saturation on perceived depth between adjacent regions in the visual field. Perception.

[42] Guibal C.R.C., Dresp B. (2004). Interaction of color and geometric cues in depth perception: When does red mean near?. Psychol. Res..

[43] Sundet J.M. (1978). Effects of colour on perceived depth. Scand. J. Psychol..

[44] Burge J., Peterson M.A., Palmer S.E. (2005). Ordinal configural cues combine with metric disparity in depth perception. J. Vis..

[45] Palmer S.E., Brooks J.L. (2008). Edge-region grouping in figure-ground organization and depth perception. J. Exp. Psychol. Hum. Percept. Perform..

[46] Predebon J., Woolley J.S. (1994). The familiar-size cue to depth under reduced-cue viewing conditions. Perception.

[47] Hochberg C.B., Hochberg J.E. (1952). Familiar size and the perception of depth. J. Psychol..

[48] Hochberg C.B., Hochberg J.E. (1953). Familiar size and subception in perceived depth. J. Psychol..

[49] Hochberg J.E., McAlister E. (1955). Relative size vs. familiar size in the perception of represented depth. Am. J. Psychol..

[50] Mershon D.H., Gogel V.C. (1975). Failure of familiar size to determine a metric for visually perceived distance. Percept. Psychophys..

[51] Gogel W.C. (1969). The absolute and relative size cues to distance. Am. J. Psychol..

[52] Gogel W.C., Hartman B.O., Harker G.S. (1957). The retinal size of a familiar object as a determiner of apparent distance. Psychol. Monogr. Gen. Appl..

[53] Gogel W.C., Mertens H.W. (1967). Perceived size and distance of familiar objects. Percept. Mot. Ski..

[54] Ittelson W.H., Ames A. (1950). Accommodation, convergence, and their relation to apparent distance. J. Psychol..

[55] Fitzpatrick V., Pasnak R., Tyer Z.E. (1982). The effect of familiar size at familiar distances. Perception.

[56] Predebon J. (1979). Role of familiar size in spatial judgments under natural viewing conditions. Percept. Mot. Ski..

[57] Predebon J. (1987). Familiar size and judgments of distance. Bull. Psychon. Soc..

[58] Predebon J. (1990). Relative distance judgments of familiar and unfamiliar objects viewed under representatively natural conditions. Percept. Psychophys..

[59] Predebon J. (1993). The familiar-size cue to distance and stereoscopic depth perception. Perception.

[60] Hosking S.G., Crassini B. (2010). The effects of familiar size and object trajectories on time-to-contact judgements. Exp. Brain Res..

[61] Ono H. (1969). Apparent distance as a function of familiar size. J. Exp. Psychol..

[62] Pirenne M.H. (1970). Optics, Painting & Photography.

[63] Epstein W. (1963). The influence of assumed size on apparent distance. Am. J. Psychol..

[64] Epstein W. (1965). Nonrelational judgments of size and distance. Am. J. Psychol..

[65] Gogel W.C. (1969). The effect of object familiarity on the perception of size and distance. Q. J. Exp. Psychol..

[66] Gogel W.C. (1969). The sensing of retinal size. Vis. Res..

[67] Gogel W.C., Mertens H.W. (1968). Perceived depth between familiar objects. J. Exp. Psychol..

[68] Ittelson W.H. (1951). Size as a cue to distance: Static localization. Am. J. Psychol..

[69] Epstein W. (1961). The known-size-apparent-distance hypothesis. Am. J. Psychol..

[70] Gogel W.C., Da Silva J.A. (1987). Familiar size and the theory of off-sized perceptions. Percept. Psychophys..

[71] McKennell A.C. (1960). Visual size and familiar size: Individual differences. Br. J. Psychol..

[72] DeLucia P.R. (2005). Image size and instructions in the perception of depth. Q. J. Exp. Psychol..

[73] Peirce J.W., Gray J.R., Simpson S., MacAskill M., Höchenberger R., Sogo H., Kastman E., Lindelov J.K. (2019). PsychoPy2: Experiments in behavior made easy. Behav. Res. Methods.

[74] Neter J., Kutner M.H., Nachtsheim C.J., Wasserman W. (1996). Applied Linear Statistical Models.

[75] Rouder J.N., Speckman P.L., Sun D., Morey R.D., Iverson G. (2009). Bayesian t tests for accepting and rejecting the null hypothesis. Psychon. Bull. Rev..

[76] Wetzels R., Matzke D., Lee M.D., Rouder J.N., Iverson G.J., Wagenmakers E. (2011). Statistical evidence in experimental psychology: An empirical comparison using 855 t tests. Perspect. Psychol. Sci..

[77] Rouder J.N., Morey R.D., Speckman P.L., Province J.M. (2012). Default Bayes factors for ANOVA designs. J. Math. Psychol..

[78] Scharff A., Palmer J., Moore C.M. (2013). Divided attention limits perception of 3-D objects shapes. J. Vis..

[79] Francis G. (2013). Replication, statistical consistency, and publication bias. J. Math. Psychol..

[80] Francis G. (2014). The frequency of excess success for articles in Psychological Science. Psychon. Bull. Rev..

[81] Francis G., Tanzman J., Matthews W.J. (2014). Excess success for psychology articles in the journal Science. PLoS ONE.

[82] Ioannidis J.P.A. (2005). Why most published research findings are false. PLoS Med..

[83] Valenti J.J., Firestone C. (2019). Finding the odd one out: Memory color effects and the logic of appearance. Cognition.

